# A comparison between patients who suffer from major depression and are treated with Esketamine – one group participates in group therapy and the other one does not

**DOI:** 10.1192/j.eurpsy.2022.1436

**Published:** 2022-09-01

**Authors:** H. Yaniv, V. Savlev

**Affiliations:** Kfar Shaul Hospital, Closed Unit, Jerusalem, Israel

**Keywords:** resistance, major depression, esketamine, group therapy

## Abstract

**Introduction:**

Major depressive disorder is present in approximately 7% of the general population. There are some patients that remain treatment-resistant - patients who were treated with two or more different medications and did not demonstrate any improvement in their mental state. These patients can be treated with a new treatment – Esketamine. The recommended Esketamine treatment protocol includes 8-treatment sessions, each session lasts about two hours. In our clinic, we added a therapy group after each treatment. The therapy group is led by two co-therapist and lasts 30 minutes. The patients are invited to share their experiences from the session, their thoughts and emotions.

**Objectives:**

The study that we will present was conducted in the Esketamine treatment unit at a psychiatric hospital. There were two groups - 1. A group whose treatment included a therapeutic group at the end of each Esketamine treatment (n=30); 2. A group whose treatments did not include a therapeutic group at the end of the Esketamine treatment (n=30).

**Methods:**

The current study examines the role of the therapeutic group. It compares between the standard treatment protocol, with and without a therapeutic group. All participants completed three questionnaires, about their emotions, three times during the treatment (before the first session, after 4 sessions and after 8 sessions).

**Results:**

We will present first results as well as vignette to demonstrate.

**Conclusions:**

The expectation is to find a better patient experience and a better insight about the clinical changes following the Esketamine treatment, in the group which participates in the therapy group

**Disclosure:**

No significant relationships.

